# Nonocarbolines A–E, *β*-Carboline Antibiotics Produced by the Rare Actinobacterium *Nonomuraea* sp. from Indonesia

**DOI:** 10.3390/antibiotics9030126

**Published:** 2020-03-17

**Authors:** Gian Primahana, Chandra Risdian, Tjandrawati Mozef, Enge Sudarman, Matthias Köck, Joachim Wink, Marc Stadler

**Affiliations:** 1 Department Microbial Drugs, Helmholtz Centre for Infection Research GmbH (HZI), Inhoffenstrasse 7, 38124 Braunschweig, Germany; Gian.Primahana@helmholtz-hzi.de (G.P.); e.sudarman@web.de (E.S.); 2 Research Center for Chemistry, Indonesian Institute of Sciences (LIPI), Kawasan Puspiptek, Serpong, 15314 Tangerang Selatan, Indonesia; tjandrawm@gmail.com; 3 Working group Microbial Strain Collection, Helmholtz Centre for Infection Research GmbH (HZI), Inhoffenstrasse 7, 38124 Braunschweig, Germany; Chandra.Risdian@helmholtz-hzi.de (C.R.); Joachim.Wink@helmholtz-hzi.de (J.W.); 4 Research Unit for Clean Technology, Indonesian Institute of Sciences (LIPI), Bandung 40135, Indonesia; 5 Alfred-Wegener-Institut, Helmholtz-Zentrum für Polar- und Meeresforschung (AWI), Am Handelshafen 12, 27570 Bremerhaven, Germany; Matthias.Koeck@awi.de

**Keywords:** *Nonomuraea* sp. 1808210 CR, rare actinobacteria, secondary metabolite, *β*-carbolines

## Abstract

During the course of our ongoing screening for novel biologically active secondary metabolites, the rare Actinobacterium, *Nonomuraea* sp. 1808210CR was found to produce five unprecedented *β*-carboline derivatives, nonocarbolines A–E (**1–5**). Their structures were elucidated from high-resolution mass spectrometry, 1D and 2D nuclear magnetic resonance spectroscopy, and the absolute configuration of **4** was determined by using the modified Mosher method. Nonocarboline B (**2**) displayed moderate antifungal activity against *Mucor hiemalis*, while nonocarboline D (**4**) exhibited significant cytotoxic activity against the human lung carcinoma cell line A-549 with the IC_50_ value of 1.7 µM.

## 1. Introduction

Due to the serious consequence and dynamic nature of antibiotic resistance in pathogens, the need for new bioactive compounds is steadily increasing, especially regarding molecules with new modes of action [1]. In the past decades, actinobacteria, particularly from the genus *Streptomyces*, have been reported to produce about two thirds of the naturally derived antibiotics in current clinical use, as well as many anticancer compounds [2]. While *Streptomyces* species appear to have been exhaustively explored [3,4], other genera belonging to the so-called “rare actinobacteria” may still serve as promising sources for novel biologically active secondary metabolites [4,5,6,7]. One of these genera is *Nonomuraea*, which has recently been reported to produce several new biologically active compounds such as the antimicrobial hypogeamycins B–D [8], nonomuric acid and 3-hydroxy deoxydaunorubicinol aglycone [9], the cytotoxic hypogeamycin A [8], and karamomycins [10]. 

In the course of our screening for novel bioactive metabolites from our rare actinobacteria collections, the strain *Nonomurea* 1808210CR was selected for further investigation because it showed significant activity (MIC 4.2 μg/mL) against *Bacillus subtilis* and some potentially new metabolites were detected in the active samples by High Performance Liquid Chromatography coupled with diode array detection and mass spectrometry (HPLC-DAD/MS). Herein, we describe the isolation, structure elucidation, and biological activities of nonocarbolines A–E (**1**–**5**), which constitute the first *β*-carbolines from this genus.

## 2. Results and Discussion

During our course for novel antibiotics from actinobacteria, significant activity by bioassay screening against *Bacillus subtilis* was detected in the extract of the strain 1808210CR. Phylogenetic 16S rRNA gene analysis showed that the aligned sequence was closely related to the DNA sequence of the type strain *Nonomuraea jabiensis* DSM 45507^T^ with 99.38% similarity (see Figures S1 and S2 in SI). The sequence was deposited in GenBank with the accession number MN 938364. The strain may represent a new species, and a polyphasic taxonomic study that will be reported elsewhere is presently ongoing.

**Figure 1:**
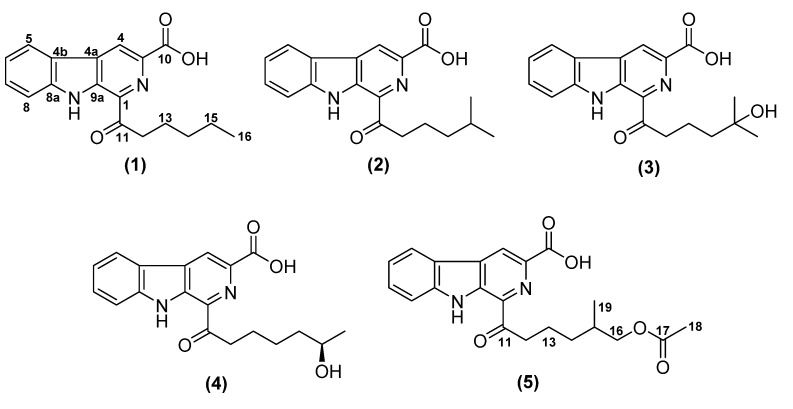
Structures of nonocarbolines A–E (**1**–**5**).

Analysis of the crude extract by HPLC-DAD/MS followed by comparison with the Dictionary of Natural Products (DNP) database (http://dnp.chemnetbase.com) suggested the presence hitherto of unknown metabolites. Accordingly, we conducted a scale-up fermentation, and subsequent chromatography of the crude extract led to the isolation of five unknown *β*-carbolines (Figure 1).

Compound **1** was isolated as a white solid. HR-ESIMS analysis of **1** exhibited molecular ion clusters at *m/z* 311.1393 [M + H]^+^ and at *m/z* 333.1208 [M + Na]^+^, indicating the molecular formulas of C_18_H_18_N_2_O_3_ (calcd. 311.1396), and C_18_H_17_N_2_O_3_Na (calcd. 333.1210) (Figures S3 and S4 in SI), respectively. Accordingly, eleven double bond equivalents (DBEs) were calculated. The characteristic UV/Vis absorption bands at 218, 264, 284 and 375 nm suggested a *β*-carboline moiety in **1** [11,12]. The complete structure of **1** was determined by 1D and 2D NMR analyses. The ^13^C NMR data confirmed the presence of 18 carbons, including one carboxylic acid (*δ*_C_ 166.3), one ketone (*δ*_C_ 203.1), five olefinic methines (*δ_H_ 7.35*–9.14; *δ*_C_ 113.3–129.2), six nonprotonated carbons, including four carbons attached to a heteroatom (*δ*_C_ 120.2–142.2), four methylene carbons (*δ*_H_ 1.37–3.41; *δ*_C_ 22.0–36.7), and one methyl group (*δ*_H_ 0.90; *δ*_C_ 13.6) (see Figures S19, S20 and S22 in SI). In addition, the ^1^H NMR spectrum of **1** in DMSO-*d*_6_ provided an NH signal at 12.27 ppm (Figure S18 in SI). The ^1^H,^1^H COSY (correlation spectroscopy) correlations (Figure S21 in SI), in conjunction with the ^1^H,^13^C HMBC correlations (Figure S23 in SI), assembled the 1,3-disubstituted *β*-carboline moiety. Furthermore, the COSY spectrum showed a series of correlations from H-12 to H-13, H-13 to H-14, H-14 to H-15, and H-15 to H-16 (see Table 1), indicating the presence of a contiguous pentyl chain, which was connected to C-1 via carbonyl C-11 from an HMBC (Heteronuclear Multiple Bond Correlation) correlation of H-13 to C-11. Since there is no direct observation of ^3^*J*_CH_ coupling in HMBC correlations from H-12 to C-1, we compared the carbon chemical shifts of the isolated compounds with those of marinacarboline B (**8**), which has a similar basic skeleton. The carbon chemical shift at C-1 of the isolated compound **1** was identical with the published data [13]. Moreover, an NOE (Nuclear Overhauser Effect) correlation between the NH and H-12 supported the location of the carbonyl side chain at C-1. Further HMBC correlations from H-4 to the carbonyl carbon C-10, together with the molecular formula, indicated that the carboxylic acid was attached to C-3. Therefore, the structure of compound **1**, for which we propose the trivial name nonocarboline A, was unambiguously determined as 1-hexanoyl-9*H*-pyrido [3,4-b] indole-3-carboxylic acid.

Nonocarboline B (**2**) was isolated as a yellow solid and its molecular formula was determined as C_19_H_20_N_2_O_3_ by the molecular ion cluster [M + H]^+^ at *m/z* 325.1548 (calcd. 325.1552) in its HR-ESIMS spectrum (see Figures S5–S8 in SI). Compared to **1**, the molecular formula of **2** includes an additional CH_2_ moiety. The ^1^H and ^13^C NMR spectral data (Figures S24–S29 in SI) of **2** were similar to those of **1**, except that the signal at *δ*_H_ 0.90 ppm (3H, t, 7.0 Hz) of **1** was replaced by an isopropyl signal (6H, d, 6.5 Hz), indicating the presence of an isohexyl chain connected to C-1 through the carbonyl C-11.

Nonocarboline C (**3**) was obtained as a yellow solid and exhibited a molecular ion peak at *m/z* 341.1495 (calcd. 341.1501) which indicated the molecular formula of C_19_H_20_N_2_O_4_ and 11 degrees of unsaturation (Figures S9–S11 in SI). The main difference in the ^1^H NMR spectrum of **3** compared to **2** is the disappearance of the proton signal of H-15. The presence of a hydroxyl group at C-15 was confirmed in the ^13^C NMR spectrum by the presence of a deshielded shifted signal of 68.8 ppm at C-15 (see Figures S30–S34 in SI).

**Figure 2:**
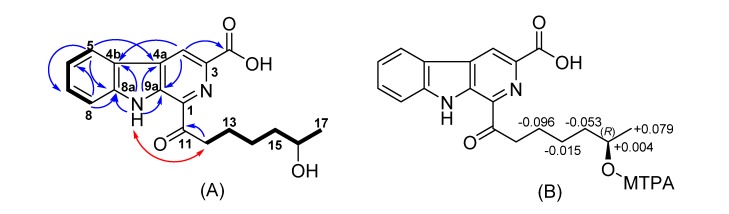
(**A**) Selected COSY (bold bond), NOESY (Nuclear Overhauser Effect Correlation SpectroscopY) red arrow) and HMBC (blue arrows) correlations of compound **4**. (**B**) Shielding effect of MTPA moiety of **4**, Δ*^S-R^* values are shown.

The molecular formula of nonocarboline D (**4**), which was isolated as a yellow solid, was determined by its HR-ESIMS to be C_19_H_20_N_2_O_4_ (11 DBE) (Figures S12–S14 in SI). The UV, mass and NMR spectra were very similar to those of **3**, indicating that **4** represents a structural isomer of **3**. The COSY spectrum showed a contiguous aliphatic chain from H-12 to H-17, bearing a hydroxyl group at C-16 due to the deshielded shift of proton signal at 3.59 ppm with the corresponding carbon at 65.7 ppm (see Figures S35–S40 in SI). The absolute configuration of **4** was determined by the modified Mosher method by esterification using (*R*)- and (*S*)-MTPA chloride to provide (*S*)-and (*R*)-MTPA esters [14,15]. The shift differences Δ*δ^S-R^* calculated between these esters (see Figures S47–S51 in SI) are depicted in Figure 2. The absolute configuration of **4** was determined to be *R*.

HR-ESIMS analysis of nonocarboline E (**5**) revealed a molecular ion peak at *m/z* 383.1608 (calcd. 383.1607) with the molecular formula C_21_H_22_N_2_O_5_ (12 DBE) (Figures S15–S17 in SI). The ^1^H NMR spectrum of **5** contained all signals of nonocarboline B (2) with additional resonances for a methylene at 3.84 and 3.92 (*δ*_C_ 68.5) and an sp^2^ carbon (*δ*_C_ 170.5). Compared to **2**, the structure of **5** included an acetoxy group which was connected to the methylene H-16 from HMBC correlations of H-16 to C-17, and of methyl-18 to C-17 (see Figures S41–S46 in SI).

**Figure 3:**
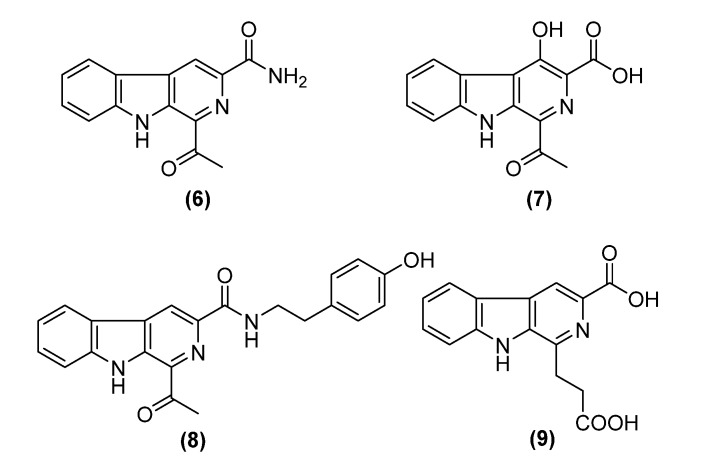
Chemical structures of selected known compounds related to this study.

The first isolated *β*-carboline alkaloid was harmaline in 1841 from the plant *Peganum harmala* [16] and since then, numerous compounds have been isolated from diverse sources, such as dichotomines A–D from plants [11], gibellamines A and B from fungi [17], and marinacarbolines A–D from bacteria [13]. Interestingly, the synthesis of a methyl ester form of **1** was reported by Chalotra et al. [18]. *β*-carbolines are known to exhibit a variety of biological activities, including antimicrobial [19,20], antitumor [21,22], antiparasitic [23,24], and antiviral effects [25]. According to our literature survey, 1,3-disubstituted *β*-carboline derivatives bearing a carbonyl moiety at C-1 and a carboxyl group at C-3 are known from plants, sponges, and fungi, while their amide-bearing analogues were reported from plants and bacteria. Of these, we show in Figure 3 stellarine A (**6**) from the marine-derived fungus *Dichotomomyces cejpii* F31-1 [26], cestrumine B (**7**) from the plant *Cestrume hediundinum* [27], and marinacarboline B (**8**) from the marine actinobacterium *Marinactinospora thermotolerans* [13].

The analogue JBIR 133 (**9**) from *Kitasatospora setae* [28] also contains a carboxylic acid group at C-3, but not at C-1 (Figure 3). However, the current study is the first report on the occurrence of *β*-carboline derivatives possessing a carboxylic acid at the C-3 position and a carbonyl moiety at C-1 that have been isolated from bacteria.

Studies on the biosynthesis of bacterial *β*-carbolines have so far been limited because only a few compounds have been isolated from these organisms. A recent study showed that the biosynthetic gene *McbB* from *Marinactinospora thermotolerans* was responsible for the biosynthesis of marinacarbolines via a Pictet–Spengler condensation of L-tryptophan and oxaloacetate [29]. Posssibly, nonocarbolines A–E are also biosynthesized in the same way by condensation of L-tryptophan and oxaloacetate, followed by chain elongation, hydroxylation, or esterification to form the final structure of nonocarbolines A–E. However, feeding studies with labeled precursors and/or elucidation of the corresponding gene cluster in our strain remain necessary to prove this hypothesis.

Nonocarbolines A–E were evaluated for antimicrobial and cytotoxic activities. Compounds **1**–**4** showed weak to moderate activity against *Bacillus subtilis*, whereas compound **5** was not active. Compound **2** was the most active derivative in our antimicrobial test panel against *Mucor hiemalis* with a minimum inhibitory concentration (MIC) at 8.3 µg/mL. Moreover, a cytotoxicity assay was conducted against six different cancer cell lines (see Table 2), and compound **4** was found to be the most active one against human lung carcinoma A-549 with an IC_50_ value of 1.7 μM, while the other metabolites showed very weak or no cytotoxic effects.

## 3. Materials and Methods

### 3.1. General Experimental Procedures

HPLC-DAD/MS analysis was performed using an amaZon speed ETD ion trap mass spectrometer (Bruker Daltonics, Bremen, Germany) in positive and negative ion modes. HPLC system (column C_18_ Acquity UPLC BEH (Waters), solvent A: H_2_O + 0.1% formic acid; solvent B: acetonitrile (ACN) + 0.1% formic acid, gradient: 5% B for 0.5 min, increasing to 100% B in 20 min, maintaining isocratic conditions at 100% B for 10 min, flow rate 0.6 mL/min, UV/Vis detection 200–600 nm). HR-ESIMS (high-resolution electrospray ionization mass spectrometry) data were recorded on a MaXis ESI TOF mass spectrometer (Bruker Daltonics) equipped with an Agilent 1260 series HPLC-UV system (Agilent Technologies, Santa Clara, CA, USA) (column C_18_ Acquity UPLC BEH (Waters), solvent A: H_2_O + 0.1% formic acid; solvent B: ACN + 0.1% formic acid, gradient: 5% B for 0.5 min, increasing 19.5 min to 100% B, holding 5 min at 100% B; flow rate 0.6 mL/min, 40 °C; DAD-UV detection at 200–600 nm). Molecular formulas were calculated using the Smart Formula algorithm (Bruker Daltonics). Fractionation and analytical RP HPLC were performed on an Agilent 1100 HPLC system. HPLC conditions: XBridge C_18_ column 100 × 2.1 mm (Waters), 3.5 μm, solvent A (5% ACN in water, 5 mmol ammonium acetate (NH_4_OAc), 0.04 mL/L CH_3_COOH); solvent B (95% ACN, 5 mmol (NH_4_OAc,) 0.04 mL/L CH_3_COOH); gradient system: from 10% B to 100% B in 30 min and maintaining at 100% for 10 min, followed by postrun from 100% to the initial condition for 10 min; flow rate 0.3 mL/min; 40 °C; fractionation was performed in 96-well plates and fractions collected every 30 s. Preparative HPLC purification was obtained with an Agilent (Santa Clara, CA, USA) 1100 series preparative HPLC system (ChemStation software (Rev. B.04.03 SP1); binary pump system; diode-array UV detector; 180-fraction collector). NMR spectra were recorded on a Bruker 700 MHz Avance III spectrometer with a 5 mm TCI cryoprobe (^1^H: 700 MHz, ^13^C: 175 MHz), locked to the deuterium signal of the solvent. Chemical shifts are given in parts per million [ppm], and coupling constants in Hertz [Hz]. UV spectra were measured on a Shimadzu (Kyoto, Japan) UV/Vis 2450 spectrophotometer using methanol (Uvasol, Merck, Darmstadt, Germany). Optical rotations were measured using Anton Paar MCP-150 Polarimeter (Graz, Austria) with 100 mm path length and sodium D line at 589 nm.

### 3.2. Strain Origin and Identification

#### 3.2.1. Sampling and Isolation of the Organism

A soil sample was collected from Malang, East Java, Indonesia. One gram of sample was heated at 60 °C for 30 min to eradicate all the vegetative cells that were present in the sample. Ten milliliters of sterile water were added to the samples, and the mixture was serially diluted (1:10, 1:100, and 1:1000). The sample was transferred on agar medium 5336 (soluble starch (10 g/L), casein (peptone Typ M) (1 g/L), K_2_HPO_4_ (0.5 g/L), MgSO_4_ × 7H_2_O (5.0 g/L), and agar (20 g/L)). The pH was adjusted to 7.3 before sterilization and supplemented with cycloheximide (100 µg/mL) as an antifungal agent [30] and incubated for 7–21 days at 30 °C.

#### 3.2.2. Analysis of 16S rRNA Sequences

Genomic DNA extraction was performed by using Invisorb Spin Plant Mini Kit (250) (Stratec Molecular, Berlin, Germany) following the manufacturer’s protocol. Amplification of 16S rRNA genes and the purification of the PCR product were performed using the methods described by Mohr et al. [31]. Two primers were employed: F27 (forward) and R1492 (reverse), the reaction volume (50 µL) was used containing water (22 µL), primers (1 µL; 10 µM each), “Jump Start Ready Mix” or JSRM (25 µl) and template DNA (1 µL). The JSRM is a mixture of JumpStart Taq DNA polymerase, 99% pure deoxynucleotides, and buffers in an optimized reaction concentration. The PCR reaction was conducted in a Mastercycler Gradient (Eppendorf, Hamburg, Germany) with the condition: initial denaturation at 95 °C (5 min); 34 cycles of denaturing at 94 °C (30 s); annealing at 52 °C (30 s); elongation at 72 °C (120 s); final elongation at 72 °C (10 min).

The PCR product was checked on the agarose gel (0.8%) and purified using the NucleoSpin^®^ Gel and PCR Clean-up Kit (Macherey-Nagel, Düren, Germany) following the manufacturer’s protocol. DNA sequencing was performed by using a 96-capillary system from Applied Biosystems (ABI), 3730xl DNA Analyzer. The primers for sequencing were F27, R518, F1100, R1100, and R1492. The 16S rRNA gene sequence was edited, and the contig was assembled and generated by BioEdit software (version 7.0.5.3) (company city country)[32]. The 16S rRNA gene sequence was deposited in GenBank with the accession number MN938364.

Identification of phylogenetic neighbors and calculation of pairwise 16S rRNA gene sequence similarities were carried out using EzTaxon-e server (http://www.ezbiocloud.net/taxonomy) [33] and the sequences of the strains were aligned using the CLUSTAL W algorithm [33] from the MEGA X software package version 10.0.5 for Windows (MEGA X, Penn State University, Pennsylvania, USA) [34]. Phylogenetic analysis was conducted using neighbor-joining [35] algorithms from MEGA X. The evolutionary distances were computed using the Kimura 2-parameter method [36]. The topologies of the inferred trees were evaluated by bootstrap analyses [37] based on 1000 replicates.

### 3.3. Scale-up Fermentation, Extraction and Isolation

A well-grown culture on an agar plate (containing glucose 4 g, yeast extract 4 g, malt extract 10 g, CaCO_3_ 10 g, agar 12 g in 1 L of deinonized water, pH adjusted to 7.2 before sterilization) was cut into small pieces (1 cm) and three pieces per flask were inoculated in a batch of thirty 250 mL Erlenmeyer flasks containing 100 mL of the medium composed of 15 g of glucose, 15 g of soybean meal, 5 g of corn steep liquor, 2 g of CaCO_3_ and 5 g of NaCl in 1 L distilled water, pH was adjusted to 7.0 before sterilization. The cultures were incubated at 37 °C on a rotary shaker (120 rpm). The strain growth was monitored by constant checking of the amount of free glucose (using Medi-Test, Macherey Nagel). The fermentation was stopped 5 days after glucose depletion. In total, 18 L of fermentation were produced in 6 batches (3 L each batch). The mycelial cake was separated from the supernatant by centrifugation (3000 rpm, 10 min). The biomass was extracted with ethyl acetate three times (1.5 L) in an ultrasonic bath at 40 °C for 30 min. After filtration and evaporation, the residue was redissolved in MeOH/H_2_O (7:3) and partitioned with n-heptane to remove the lipophilic components. The methanol layer was evaporated until the water phase and extracted with ethyl acetate three times (250 mL each). Organic layers were combined, dried over anhydrous sodium sulfate and evaporated to afford 470 mg residue which was subjected to flash chromatography (Grace Reveleris^®^, Maryland, USA) (silica cartridge 24 g, solvent A: DCM, solvent B: acetone, gradient: 2% B isocratic 1 min, from 2% B to 9% B in 11 min, 9% B isocratic 6 min, from 9% B to 30% B in 6 min and to 100% B in 5 min). Thirteen fractions were collected, the solvent was evaporated and fraction 1 (45 mg) was further purified by preparative reversed phase (RP) HPLC (Nucleodur Phenyl-Hexyl, 5 µm column, 250 × 21.2 mm (Macherey-Nagel), solvent A: water, solvent B: ACN, flow rate 20 mL/min and UV detection at 210, 280, and 360 nm, gradient: 40% B isocratic for 2 min, from 40% B to 63% B in 5 min and 63% B isocratic for 43 min) to yield compound **1** (3.61 mg, *t*_R_ = 13.1 min) and compound **2** (2.67 mg, *t*_R_ = 15.1 min). Fraction 3 (6.2 mg) was purified using a gradient system starting from 50% B, isocratic for 3 min and increasing to 75% B in 42 min, then to 100% B in 10 min followed by washing at 100% B in 5 min to afford compound **5** (1.67 mg, *t*_R_ = 27.7 min). Fraction 10 (26.7 mg) was purified under isocratic conditions at 37% B for 50 min to afford compound **3** (2.4 mg, *t*_R_ = 12.6 min).

Amberlite XAD-16 adsorber resin (Rohm and Haas, Frankfurt, Germany, 3% *v*/*v*) was added to the supernatant and stirred for 2 h. The XAD resin was collected by sieving and washed with distilled water, then extracted in a glass column (5 × 40 cm) with acetone (three portions of 500 mL each, flow rate 15 mL/min). The combined acetone was evaporated to the water phase (250 mL) and extracted with ethyl acetate three times. The organic layers were combined, dried over anhydrous sodium sulfate and evaporated. The lipophilic component was eliminated by partitioning over MeOH/n-heptane to afford 370 mg crude extract. The crude extract was subsequently subjected to Sephadex LH-20 column chromatography (Pharmacia Biotec, Piscataway, NJ, USA) (column 3 × 83 cm, flow rate 3.8 mL/min, UV detection at 280 nm, with methanol as mobile phase). Six fractions were collected and evaporated to dryness. Fraction 2 (131 mg) was further purified (3 times) by applying stepwise gradient starting with isocratic at 37% for 34 min then increased to 55% B in 3 min and subsequently increasing the percentage of solvent B to 70% in 14 min to afford compound **4** (3.2 mg).

Nonocarboline A (**1**): white solid; UV λ_max_ MeOH (log *ε*) 218 (4.05), 265 (3.86), 284 (3.95), 375 (3.30) nm; NMR data (^1^H: 700 MHz, ^13^C 176 MHz, DMSO-*d*_6_) see Table 1; HR-ESIMS: [M + H]+ *m/z* 311.1393, calcd. 311.1396 for C_18_H_19_N_2_O_3_, [M + Na]+ *m/z* 333.1208, calcd. 333.1215 for C_18_H_18_N_2_NaO_3_, [2M + Na]+ *m/z* 643.2509, calcd. 643.2527 for C_36_H_36_N_4_NaO_6_, *t*_R_ = 11.4 min.

Nonocarboline B (**2**): yellow solid; UV λ_max_ MeOH (log *ε*) 218 (4.07), 265 (3.76), 284 (3.86), 375 (3.22) nm; NMR data (^1^H: 700 MHz, ^13^C 176 MHz, DMSO-*d*_6_) see Table 1; HR-ESIMS: [M + H]+ *m/z* 325.1548, calcd. 325.1552 for C_19_H_21_N_2_O_3_, [M + Na]+ *m/z* 347.1365, calcd. 347.1372 for C_19_H_20_N_2_NaO_3_, [2M + Na]+ *m/z* 671.2826, calcd. 671.2840 for C_38_H_40_N_4_NaO_6_, *t*_R_ = 12.2 min.

Nonocarboline C (**3**): yellow solid; UV λ_max_ MeOH (log *ε*) 218 (4.29), 265 (4.09), 284 (4.16), 375 (3.48) nm; NMR data (^1^H: 700 MHz, ^13^C 176 MHz, DMSO-*d*_6_) see Table 1; HR-ESIMS: [M + H]^+^ *m/z* 341.1495, calcd. 341.1501 for C_19_H_21_N_2_O_4_, [M + Na]^+^ *m/z* 363.1314, calcd. 363.1321 for C_19_H_20_N_2_NaO_4_, [2M + Na]+ *m/z* 703.2721, calcd. 703.2738 for C_38_H_40_N_4_NaO_8_, *t*_R_ = 8.5 min.

Nonocarboline D (**4**): yellow solid; ${\left[\alpha \right]}_{D}^{20}$ +55 (*c* 0.17, MeOH); UV λ_max_ MeOH (log *ε*) 218 (4.29), 265 (4.37), 284 (4.45), 375 (3.79) nm; NMR data (^1^H: 700 MHz, ^13^C 176 MHz, DMSO-*d*_6_) see Table 1; HR-ESIMS: [M + H]^+^ *m/z* 341.1502, calcd. 341.1501 for C_19_H_21_N_2_O_4_, [M + Na]^+^ *m/z* 363.1314, calcd. 363.1321 for C_19_H_20_N_2_NaO_4_, [2M + Na]+ *m/z* 703.2727, calcd. 703.2738 for C_38_H_40_N_4_NaO_8_, *t*_R_ = 8.7 min.

Nonocarboline E (**5**): yellow solid; ${\left[\alpha \right]}_{D}^{20}$ + 12 (*c* 0.17, MeOH); UV λ_max_ MeOH (log *ε*) 218 (4.37), 264 (4.17), 284 (4.27), 375 (3.62) nm; NMR data (^1^H: 700 MHz, ^13^C 176 MHz, DMSO-*d*_6_) see Table 1; HR-ESIMS: [M + H]^+^ *m/z* 383.1608, calcd. 383.1607 for C_21_H_23_N_2_O_5_, [M + Na]^+^ *m/z* 405.1418, calcd. 405.1426 for C_21_H_22_N_2_NaO_5_, [2M + Na]^+^ *m/z* 787.2934, calcd. 787.2950 for C_42_H_44_N_4_NaO_10_, *t*_R_ = 10.6 min.

### 3.4. Preparation of (R)-and (S)-MTPA Ester Derivatives of 4

Compound **4** (1 mg) was dissolved in 1 mL of deuterated pyridine and transferred into two NMR tubes (each 0.5 mL). (*R*)-(−)-α-Methoxy-α-(trifluoromethyl)phenylacetyl chloride ((*R*)-MTPA-Cl) (2 µL) was added into one NMR tube, and another NMR tube was added with (*S*)-(+)-α-Methoxy-α-(trifluoromethyl)phenylacetyl chloride ((*S*)-MTPA-Cl). After 1 h, the reaction mixtures were measured for ^1^H NMR and ^1^H,^1^H-COSY.

### 3.5. Antimicrobial Assay

Minimum inhibitory concentrations were determined by a serial dilution assay in 96-well plates according to our standard protocols [38]. Twenty microliter aliquots of compounds **1**–**5** with an initial concentration of 1 mg/mL (the final concentration in the first well is 67 μg/mL) were tested against three different Gram-positive bacteria (*Bacillus subtilis*, *Staphylococcus aureus* Newman, and *Mycobacterium smegmatis*), five Gram-negative bacteria (*Acinetobacter baumanii*, *Citrobacter freundii*, *Escherichia coli* wild type, *Escherichia coli* strain acrB, and *Pseudomonas aeruginosa*) and three fungi (*Candida albicans*, *Mucor hiemalis* and *Pichia anomala*,) with methanol as a negative control. Oxytetracycline, ciprofloxacin, and kanamycin were used as positive controls against Gram-positive and Gram-negative bacteria, whereas nystatin was used against fungi.

### 3.6. Cytotoxicity Activity

Cytotoxicity (IC_50_) of compounds **1**–**5** was determined against seven human cancer cell lines by using an MTT assay according to an established procedure [39]. The cell lines were cultured in DMEM (Gibco, ThermoFisher Scientific, Hilden, Germany) and RPMI media (Lonza, Cologne, Germany) for MCF-7. All cell lines were supplemented with 10% fetal bovine serum (Gibco) and incubated under 10% CO_2_ at 37 °C. Epothilone B was used as a positive control and methanol as a negative control.

## 4. Conclusions

The current study shows that rare Actinobacteria are still promising sources for novel bioactive metabolites, since five new *β*-carboline alkaloids have been discovered from a strain that probably represents a new species of the rare and underexploited genus *Nonomuraea*. Even though the preliminary biological characterization of the novel molecules did not give any hints on their potential for drug development because their effects in biological systems were rather moderate and not particularly selective, the outcome of this work should give encouragement to continue the search for novel producer strains, in particular in hitherto underexploited geographic areas like Indonesia.

## Figures and Tables

**Table 1:** NMR data of compounds **1**–**5** in DMSO-*d*_6_ (^1^H, 700.4 MHz, ^13^C, 176.1 MHz).

Pos.	1		2		3		4		5		Mar. B [13]
	*δ*_H_, m (*J* in Hz)	*δ*_C_, Type	*δ*_H_, m (*J* in Hz)	*δ*_C_, Type	*δ*_H_, m (*J* in Hz)	*δ*_C,_, Type	*δ*_H_, m (*J* in Hz)	*δ*_C,_, Type	*δ*_H_, m (*J* in Hz)	*δ*_C,_, Type	*δ*_C,_, Type
1	-	134.8, C	-	134.8, C	-	134.6, C	-	134.6, C	-	134.3, C	133.9, C
2	-	-	-	-	-	-	-	-	-	-	-
3	-	136.5, C	-	136.4, C	-	138.0, C	-	138.8, C	-	134.5, C	138.8, C
4	9.14, s	120.8, CH	9.14, s	120.8, CH	9.05, s	120.2, CH	9.10, s	120.5, CH	9.04, s	120.2, CH	117.8, CH
4a	-	131.4, C	-	131.4, C	-	131.2, C	-	131.3, C	-	131.2, C	131.8, C
4b	-	120.2, C	-	120.2, C	-	120.5, C	-	120.3, C	-	120.5, C	120.2, C
5	8.44, d (7.5)	122.1, CH	8.44, d (7.8)	122.1, CH	8.38, d (7.7)	120.3, CH	8.41, d (7.8)	122.0, CH	8.37, d (7.5)	121.9, CH	122.2, CH
6	7.35, t (7.5)	120.9, CH	7.35, t (7.8)	120.9, CH	7.32, t (7.7)	121.9, CH	7.32, t (7.8)	120.6, CH	7.31, t (7.5)	120.4, CH	120.7, CH
7	7.63, t (7.5)	129.2, CH	7.63, t (7.8)	129.2, CH	7.60, t (7.7)	128.9, CH	7.61, t (7.8)	129.0, CH	7.59, t (7.5)	128.8, CH	129.2, CH
8	7.83, d (7.5)	113.3, CH	7.83, d (7.8)	113.3, CH	7.81, d (7.7)	113.1, CH	7.81, d (7.8)	113.2, CH	7.81, d (7.5)	113.1, CH	113.2, CH
8a	-	142.2, C	-	142.2, C	-	142.1, C	-	142.2, C	-	142.1, C	142.3, C
9	12.27, s	-	12.28, s	-	12.08, s	-	12.17, s	-	12.06, s	-	-
9a	-	135.0, C	-	135.0, C	-	134.4, C	-	134.8, C	-	134.6, C	134.7, C
10	-	166.3, C	-	166.3, C	-	167.1, C	-	166.8, C	-	167.1, C	163.7, C
11	-	203.1, C	-	203.0, C	-	203.4, C	-	203.2, C	-	203.2, C	202.2, C
12	3.41, t (7.4)	36.7, CH_2_	3.40, t (7.2)	36.9, CH_2_	3.39, t (7.0) *^ov^*	37.3, CH_2_	3.41, t (7.2)	36.8, CH_2_	3.40, t (7.0)	36.9, CH_2_	-
13	1.75, m	23.2, CH_2_	1.76, m	21.4, CH_2_	1.79, m	18.8, CH_2_	1.74, m	23.8, CH_2_	1.83; 1.73, m	20.9, CH_2_	-
14	1.39, m *^ov^*	30.9, CH_2_	1.30, m	38.1, CH_2_	1.48, m	43.2, CH_2_	1.46, m	25.1, CH_2_	1.48; 1.27, m	32.5, CH_2_	-
15	1.37, m *^ov^*	22.0, CH	1.61, m	27.3,CH	-	68.8, C	1.38, m	39.1, CH_2_	1.81, m	32.0, CH	-
16	0.90, t (7.0)	13.6, CH_3_	0.90, d (6.5)	22.4, CH_3_	1.10, s	29.3, CH_3_	3.59, m	65.7, CH	3.92; 3.84, dd (10.5; 6.0)	68.5, CH_2_	-
17	-	-	0.90, d (6.5)	22.4, CH_3_	1.10, s	29.3, CH_3_	1.04, d (6.0)	23.6, CH_3_	-	170.5, C	-
18							-	-	2.00, s	20.6, CH_3_	-
19							-	-	0.92, d (6.6)	16.6, CH_3_	-

*ov*: overlapping, compound **3** (with water signal), chemical shifts were assigned from the ^1^H,^13^C-HSQC spectrum; Mar. B: marinacarboline B.

**Table 2:** Antimicrobial and cytotoxicity activities of **1**–**5**.

Microorganism	[1]	[2]	[3]	[4]	[5]	Reference [µg/mL]
	MIC [µg/mL]					
*Escherichia coli acrB* JW0451-2	-	66.7	-	**-**	-	8.33 ^a^
*Bacillus subtilis* DSM 10	66.7	33.4	66.7	16.7	-	0.52 ^b^
*Staphylococcus aureus* Newman	-	66.7	-	66.7	-	0.52 ^a^
*Mycobacterium smegmatis* ATCC 700084	-	66.7	-	-	-	0.52 ^c^
*Mucor hiemalis* DSM 2656	33.4	8.3	-	-	-	4.20 ^d^
**Cell line**	**IC_50_ [µM]**					**Ref ^e^ [µM]**
mouse fibroblast L-929	35.4	89.5	-	-	-	1.1 × 10^−3^
HeLa cells KB-3.1	-	51.6	77.4	2.3	-	5.9 × 10^−5^
human breast adenocarcinoma MCF-7	-	-	-	77.4	-	-
human lung carcinoma A-549	-	-	-	1.7	-	-
human prostate cancC-3	-	-	-	10.6	-	-
ovarian carcinoma SKOV-3	-	-	-	17.1	-	2.9 × 10^−4^
squamous cell carcinoma A-431	-	-	-	58.1	-	6.7 × 10^−5^

^a^ Oxytetracycline; ^b^ Ciprofloxacin; ^c^ Kanamycin; ^d^ Nystatin; ^e^ Epothilone B; - (not active).

## Supplementary Materials

The following are available online at https://www.mdpi.com/2079-6382/9/3/126/s1, Figure S1: *Nonomuraea* sp. 1808210CR on GYM agar plate. Figure S2: Phylogenetic tree of *Nonomuraea* sp. 1808210CR, Figure S3: HPLC-DAD-MS chromatogram of compound **1**, Figure S4: HR-ESIMS chromatogram of compound **1**, Figure S5: UV/Vis spectrum of compound **1** in MeOH, Figure S6: HPLC-DAD-MS chromatogram of compound **2**, Figure S7: HR-ESIMS chromatogram of compound **2**, Figure S8: UV/Vis spectrum of compound **2** in MeOH, Figure S9: HPLC-DAD-MS chromatogram of compound **3**, Figure S10: HR-ESIMS chromatogram of compound **3**, Figure S11: UV/Vis spectrum of compound **3** in MeOH, Figure S12: HPLC-DAD-MS chromatogram of compound **4**, Figure S13: HR-ESIMS chromatogram of compound **4**, Figure S14: UV/Vis spectrum of compound **4** in MeOH, Figure S15: HPLC-DAD-MS chromatogram of compound **5**, Figure S16: HR-ESIMS chromatogram of compound **5**, Figure S17: UV/Vis spectrum of compound **5** in MeOH, Figure S18: ^1^H NMR spectrum of **1** in DMSO-*d_6_*, Figure S19: ^13^C NMR spectrum of **1** in DMSO-*d_6_*, Figure S20: DEPT NMR of **1** in DMSO-*d_6_*, Figure S21: ^1^H,^1^H COSY of **1** in DMSO-*d_6_*, Figure S22: ^1^H,^13^C HSQC NMR of **1** in DMSO-*d_6_*, Figure S23: ^1^H,^13^C HMBC NMR of **1** in DMSO-*d_6_*, S24: ^1^H NMR spectrum of **2** in DMSO-*d_6_*, Figure S25: ^13^C NMR spectrum of **2** in DMSO-*d_6_*, Figure S26: DEPT NMR of **2** in DMSO-*d_6_*, Figure S27: ^1^H,^1^H COSY of **2** in DMSO-*d_6_*, Figure S28: ^1^H,^13^C HSQC NMR of **2** in DMSO-*d_6_*, Figure S29: ^1^H,^13^C HMBC NMR of **2** in DMSO-*d_6_*, Figure S30: ^1^H NMR spectrum of **3** in DMSO-*d_6_*, Figure S31: ^13^C NMR spectrum of **3** in DMSO-*d_6_*, Figure S32: ^1^H,^1^H COSY of **3** in DMSO-*d_6_*, Figure S33: ^1^H,^13^C HSQC NMR of **3** in DMSO-*d_6_*, Figure S34: ^1^H,^13^C HMBC NMR of **3** in DMSO-*d_6_*, Figure S35: ^1^H NMR spectrum of **4** in DMSO-*d_6_*, Figure S36: ^13^C NMR spectrum of **4** in DMSO-*d_6_*, Figure S37: DEPT NMR of **4** in DMSO-*d_6_*, Figure S38: ^1^H,^1^H COSY of **4** in DMSO-*d_6_*, Figure S39: ^1^H,^13^C HSQC NMR of **4** in DMSO-*d_6_*, Figure S40: ^1^H,^13^C HMBC NMR of **4** in DMSO-*d_6_*, Figure S41: ^1^H NMR spectrum of **5** in DMSO-*d_6_*, Figure S42: ^13^C NMR spectrum of **5** in DMSO-*d_6_*, Figure S43: DEPT NMR of **5** in DMSO-*d_6_*, Figure S44: ^1^H,^1^H COSY of **5** in DMSO-*d_6_*, Figure S45: ^1^H,^13^C HSQC NMR of **5** in DMSO-*d_6_*, Figure S46: ^1^H,^13^C HMBC NMR of **5** in DMSO-*d_6_*, Figure S47: ^1^H NMR reaction mixture of (*R*)-nonocarboline D-MTPA ester in pyridine-*d_5_*, Figure S48: ^1^H,^1^H COSY NMR spectrum of reaction mixture of (*R*)-nonocarboline D-MTPA ester in pyridine-*d_5_*, Figure S49: ^1^H NMR reaction mixture of (*S*)-nonocarboline D-MTPA ester in pyridine-*d_5_*, Figure S50: ^1^H,^1^H COSY NMR spectrum of reaction mixture of (*S*)-nonocarboline D-MTPA ester in pyridine-*d_5_*, Figure S51: Rection scheme and ^1^H NMR chemical shifts after Mosher’s reaction, in the supplementary materials.
